# Antibiotic resistance pattern and virulence genes content in avian pathogenic *Escherichia coli* (APEC) from broiler chickens in Chitwan, Nepal

**DOI:** 10.1186/s12917-018-1442-z

**Published:** 2018-03-27

**Authors:** Manita Subedi, Himal Luitel, Bhuminanda Devkota, Rebanta Kumar Bhattarai, Sarita Phuyal, Prabhat Panthi, Anil Shrestha, Dhiraj Kumar Chaudhary

**Affiliations:** 1 Department of Drug Administration, Government of Nepal, Bijulibazar, Kathmandu, Nepal; 2 Center for Biotechnology, Agriculture and Forestry University, Rampur, Chitwan Nepal; 3 Department of Veterinary Microbiology and Parasitology, Agriculture and Forestry University, Rampur, Chitwan Nepal; 4 Department of Veterinary Science & Animal Husbandry, Himalayan College of Agriculture Sciences and Technology, Kathmandu, Nepal; 5 Department of Microbiology, National College, Kathmandu, Nepal; 6 Department of Microbiology, Balkumari College, Bharatpur, Chitwan Nepal; 7 Department of Microbiology, Prithu Technical College, Institute of Agriculture and Animal Science, Tribhuvan University, Lamahi, Dang, Nepal

**Keywords:** Avian pathogenic *Escherichia coli* (APEC), Antimicrobial resistance, Virulence gene, Broiler chicken, PCR

## Abstract

**Background:**

Avian pathogenic *Escherichia coli* (APEC) are causative agent of extraintestinal infections, collectively known as colibacillosis, which results significant losses in poultry industries. The extraintestinal survival of *E. coli* is facilitated by numerous virulence factors which are coded by virulence genes. This study was conducted to find out the pattern of antibiotic resistance and virulence genes content in the APEC strains isolated from broiler chickens at National Avian Disease Investigation Laboratory and Veterinary Teaching Hospital, Rampur, Chitwan, Nepal.

**Results:**

A total of 50 *E. coli* strains were isolated from 50 colibacillosis suspected broiler chickens. Out of 50 isolates of *E. coli*, 47 (94%) showed resistant to three or more antimicrobials. The highest levels (22%) of multidrug-resistant *E. coli* were observed for five different types of antimicrobials. Antibiogram profiles of 50 *E. coli* strains showed the maximum resistance to ampicillin (98%), followed by co-trimoxazole (90%), and doxycycline (62%). The highest intermediate resistance was shown by colistin (50%) and the highest sensitivity was against amikacin (84%), followed by nitrofurantoin (55%). Based on the genetic criteria, 45 (90%) *E. coli* isolates were considered as pathogenic (APEC) which contained more than five virulence genes. Out of total APEC genes detected, we found the combination of *iss, iucD, hlyF, ompT, iroN,* and *iutA* genes were mostly associated with the APEC and additionally, to some lesser extent *irp2, papC, Cva/cvi,* and *tsh* genes showed the critical role for virulent traits of APEC strains.

**Conclusion:**

In this study, high prevalent of antimicrobial resistant pattern was found with avian pathogenic *E. coli* strains isolated from broiler chickens. To our knowledge, this is the first molecular analysis which confirmed the prevalence of APEC strains in poultry sector in Nepal. These finding suggest the need of surveillance and intervention system to control misuse of antibiotics and APEC outbreak in the poultry farm.

**Electronic supplementary material:**

The online version of this article (10.1186/s12917-018-1442-z) contains supplementary material, which is available to authorized users.

## Background

*Escherichia coli* are considered as normal inhabitant of gastrointestinal tract of man and animals. It is also a part of normal intestinal microflora in bird [1]. But, certain pathogenic strains of *E. coli* invade different organs of bird and causes pericarditis, air sacculitis, perihepatitis, peritonitis, and other extraintestinal diseases, collectively termed as colibacillosis [1, 2]. Colibacillosis may be localized or systemic and caused by avian pathogenic *Escherichia coli* (APEC) [3]. The pathogenic ability of *E. coli* strain is facilitated by broad range of virulence factors which are coded by virulence-associated genes (*iutA, iss, papC, iucD, tsh, irp-2, ompT, hlyF, iron, cva/cvi,* and *astA*). According to the genetic criteria, the pathogenicity of APEC strain is determined by presence of at least five virulence genes [1]. Some human and avian extraintestinal pathogenic *E.coli* (ExPEC) has similar phylogenic backgrounds and shares similar virulence genes possessing zoonotic risk [4]. The sequencing of the genome of the APEC strain O1:K1:H7 revealed a high similarity to the genome of human uropathogenic *E. coli* (UPEC) and neonatal meningitis *E. coli* (NMEC) [5].

Antibiotics are commonly used in the poultry farm to circumvent the challenges arise due to APEC strains. In many countries including Nepal, there is excessive use of antibiotics in poultry industries. Studies have reported that antibiotics have been used in chicken broilers as growth promoter and disease preventive measures [6, 7]. However, utilization of antimicrobials in food producing animals have created several adverse effects, such as changes in intestinal micro flora, presence of residual antibiotics in food products, impact on public environment, and emergence of antimicrobial resistance in microorganisms [8, 9]. Multiple antibiotic resistant microbes have challenged in the treatment of zoonotic diseases and its transmission from animal to human have led to the threatening situation in health sectors [10].

Poultry industries are emerging rapidly in the developing countries like Nepal. Chitwan, Kathmandu, and Kaski districts are the major areas of poultry farms in Nepal [6]. Chitwan district solely produces 68% and 10% of total eggs and chicken meats, respectively in the country [11]. But, outbreaks of different types of diseases in the poultry farms cause significant economic losses. Among the diseases reported, the outbreak of colibacillosis is one of the major problems of poultry industries [12]. Colibacillosis among broiler chicken is endemic in Nepal. Studies have reported that the prevalence of colibacillosis ranges from 10 to 60% in Chitwan district. The frequent incidence of avian diseases has been increased tremendously [11, 13]. In the context of Nepal, only few literatures are available regarding assessment and investigation of APEC disease. In addition, the trend of pathological investigations of colibacillosis in Nepal is based on clinical symptoms and isolation of *E. coli* from fecal samples [11]. These conventional approaches of investigations always pose the risk of reporting non-pathogenic *E. coli*. Furthermore, studies on virulence genes and molecular detection of APEC strains from broiler chicken of Nepal have not been reported yet. Therefore, this study was conducted to find the pattern of antibiotic resistance and virulence genes content among APEC strains isolated from broiler chickens.

## Methods

### Sample collections, bacterial isolation and identification

Fifty liver samples were collected from 50 colibacillosis suspected broiler chickens which were attended from May 2016 to March 2017 for routine diagnosis at National Avian Disease Investigation Laboratory (NADIL) and Veterinary Teaching Hospital, Rampur, Nepal. For isolation of *E. coli* strains, swab from the liver sample was aseptically streaked directly on the MacConkey agar (HiMedia, M081) and incubated aerobically at 37 °C for 24 h. The pure colonies were further streaked in the eosine methylene blue (EMB) agar (HiMedia, M317) and incubated overnight at 37 °C. Colonies with the green metallic sheen on EMB agar were suspected as *E. coli* strains and the further confirmation was done by following the standard microbiological techniques which include studies of colony morphology, Gram staining, and biochemical tests (indole, methyl red, Voges-Proskauer, citrate, catalase, oxidase, and motility indole ornithinase test) [14, 15].

### Antibiotic susceptibility testing

Antibiotic susceptibility test of the isolates was performed following modified Kirby-bauer disk diffusion method as recommended by Clinical and Laboratory Standards Institute (CLSI) [16]. The antibiotics used in this study were amikacin (AK), nitrofurantoin (NIT), ciprofloxacin (CIP), levofloxacin (LE), gentamicin (GEN), ampicillin (AMP), co-trimoxazole (COT), doxycycline hydrochloride (DO), and colisitin (CL). These antibiotics were selected due to their extensive consumption in the poultry feed and treatment of colibacillosis and other avian diseases [7, 11, 13]. All the antibiotic discs used in this study were purchased from Himedia, India. For quality control, *E coli* ATCC 25922 and *Pseudomonas aeruginosa* ATCC 27853 were used as reference strains.

### Detection of virulence genes

Isolated *E. coli* strains were investigated for the presence of eleven virulence genes (*iutA, iss, papC, iucD, tsh, irp-2, ompT, hlyF, iron, cva/cvi,* and *astA*) which are associated with colibacillosis. For the detection of virulence genes, genomic DNA was extracted from pure cultures of *E. coli* grown overnight in the MacConkey agar at 37 °C by using the DNeasy Blood and Tissue Kit (Qiagen, catalogue no. 69506). The quality of genomic DNA was checked by gel electrophoresis and measuring absorbance at *A*_*260*_/*A*_*280*_ and *A*_*260*_/*A*_*230*_ ratios using the MaestroNano spectrophotometer (MaestroGen; Model name: MN-913). The conventional PCR was used to amplify the virulence genes. The primers used for amplification were those described previously (Table 1) [17, 18]. The PCR was performed in 25 μL volume containing 12.5 μL Hot start Taq 2X master mix (BioLab Inc., New England), 1 μL each primer, 2 μL DNA template, and 8.5 μL nuclease free water. The PCR amplifications were conducted in MultiGene OptiMax Thermal Cycler (Labnet International, Inc., North America) and the cycling conditions were identical for all the samples as follows: 94 °C for 4 min; 35 cycles of 30 s at 94 °C, 1 min at 60 °C, and 2 min at 68 °C; and 72 °C for 7 min. The amplicons were analyzed by agarose gel electrophoresis with 1.5% agarose gel (Sigma-Aldrich, A4718) prepared in 1× TBE buffer (ThermoFisher Scientific, B52). All the PCR products were stained with ethidium bromide. After electrophoresis, the bands were visualized and photographed under UV light. The amplified product was considered to contain virulence gene if it produced band of the expected size.

**Table 1:** Primer sets for detection of target virulence genes from avian pathogenic *Escherichia coli* (APEC) isolates

Genes	Primer Sequence (5′–3′)	Amplicon size (bp)
*iutA*	F: GGCTGGACATCATGGGAACTGG R: CGTCGGGAACGGGTAGAATCG	302
*iss*	F: CAGCAACCCGAACCACTTGATG R: AGCATTGCCAGAGCGGCAGAA	323
*papC*	F: TGATATCACGCAGTCAGTAGC R: CCGGCCATATTCACATAA	501
*iucD*	F: ACAAAAAGTTCTATCGCTTCC R: CCTGATCCAGATGATGCTC	714
*tsh*	F: ACTATTCTCTGCAGGAAGTC R: CTTCCGATGTTCTGAACGT	824
*irp-2*	F: AAGGATTCGCTGTTACCGGAC R: AACTCCTGATACAGGTGGC	413
*ompT*	F: TCATCCCGGAAGCCTCCCTCACTACTAT R: TAGCGTTTGCTGCACTGGCTTCTGATAC	496
*hlyF*	F: GGCCACAGTCGTTTAGGGTGCTTACC R: GGCGGTTTAGGCATTCCGATACTCAG	450
*iroN*	F: AATCCGGCAAAGAGACGAACCGCCT R:GTTCGGGCAACCCCTGCTTTGACTTT	553
*cva/cvi*	F: TGGTAGAATGTGCCAGAGCAAG R: GAGCTGTTTGTAGCGAAGCC	1181
*astA*	F: TGCCATCAACACAGTATATCC R: TCAGGTCGCGAGTGACGGC	116

### Statistical analysis

Data entry and analysis were done using the program Microsoft Office Excel 2010 and *Chi*-square test was performed. The *p*-value was calculated and considered significant only when it was less than 0.05. The Multiple Antibiotic Resistance (MAR) index was calculated as a/b [19], where ‘a’ is the number of resistance antibiotics and ‘b’ is the number of antibiotics used.

## Results

**Fig. 1:**
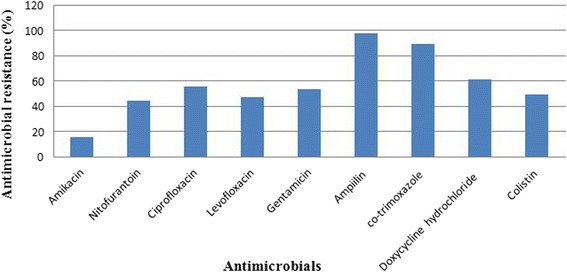
Antimicrobial resistance pattern of tested antibiotics among *E. coli* strains isolated from colibacillosis suspected broiler chickens

A total of 50 *E. coli* strains were isolated from 50 liver swab samples of colibacillosis suspected broiler chickens. The antibiogram profile of *E. coli* isolates showed highest resistance to ampicillin (98%) and least resistance to amikacin (16%) (Fig. 1). Out of 50 *E. coli* isolates, 47 (94%) isolates were resistant to three or more antibiotics. The MAR index analysis showed 94% of *E. coli* isolates had MAR index value of > 0.2 and 6% had MAR index value of ≤0.2. The proportions of isolates with the MAR index values of 0.3, 0.4, 0.5, and 0.6 were 31%, 21%, 22%, and 20%, respectively. There was no significant association of prevalence of antibiotic resistant strains with the type of *E. coli* strains (*P* > 0.05).

Based on the genetic criteria for the pathogenicity, isolates containing at least five virulence genes were considered as the APEC strains and isolates containing less than five virulence genes were considered as the avian non-pathogenic *Escherichia coli* (non-APEC) strains. Out of 50 *E. coli* isolates, 45 (90%) isolates were found to be APEC strains and 5 (10%) isolates were found to be non-APEC strains (Table 2). Among 50 *E. coli* strains, 7 strains contained all the eleven virulence genes, 14 strains contained ten virulence genes, 15 strains contained nine virulence genes, 5 strains contained eight virulence genes, 2 strains contained seven virulence genes, 2 strains contained five virulence genes, 4 strains contained 4 virulence genes, and 1 strain contained 3 virulence genes. The virulence genes *iss, ompT, hlyF,* and *iroN* were detected from all the 45 APEC strains. The frequencies of *iucD, astA, iutA, irp-2,* and *tsh* genes in the APEC strains were 97.8%, 95.6%, 82.2%, 73.3%, and 62.2%, respectively. The presence of virulence genes *cva/cvi* and *papC* showed the lowest frequency among APEC strains (Table 2; Additional file 1: Figure S1). High frequency of the five virulence genes (*iss, ompT, hlyF, iroN*, and *iucD*) was observed among the APEC isolates compared to the non-APEC. Statistical analysis showed that the distribution of virulence genes was significantly associated with the APEC and the non-APEC strains of *E. coli* (*P* < 0.05).

**Table 2:** Virulence gene frequency in avian pathogenic *Escherichia coli* (APEC) and avian non-pathogenic *Escherichia coli* (Non-APEC) isolates

Genes	APEC isolates (*n* = 45) n(%)	Non-APEC isolates (*n* = 5) n(%)	Total (*n* = 50) n(%)
*iutA*	37(82.2)	1 (20)	38 (76)
*iss*	45 (100)	0 (0)	45 (90)
*papC*	25 (55.6)	0 (0)	25 (50
*iucD*	44 (97.8)	3 (60)	47 (94)
*tsh*	28 (62.2)	0 (0)	28 (56)
*irp-2*	33 (73.3)	3 (60)	36 (72)
*ompT*	45 (100)	4 (80)	49 (98)
*hlyF*	45 (100)	3 (60)	48 (96)
*iroN*	45 (100)	1 (20)	46 (92)
*cva/cvi*	26 (57.8)	2 (40)	28 (56)
*astA*	43 (95.6)	2 (40)	45 (90)

## Discussion

Commercialized poultry industries consume wide range of antibiotics for disease prevention and growth promotion [20]. Globally, the consumption of antimicrobials in food animal production is projected to rise by 67%, by 2030. The antimicrobial use in the chicken is expected to rise by 129%, by 2030 in the Asia-Pacific region [21]. Avian colibacillosis is the major disease in chicken which has been reported by several previous studies [3, 12, 22]. The therapeutic use of antimicrobials in poultry industries is considered as efficient control measure for colibacillosis. However, evolution of multidrug resistant strains along with the transmission of resistance genes has created challenges in reducing risk of APEC infections. In Nepal, the accurate data of prevalence of multi-antibiotic resistant APEC strains is hardly documented. Next, molecular based studies for the detection of virulence genes associated with colibacillosis are lacking. Therefore, this study has attempted to find the multiple antibiotic resistant patterns and detects eleven virulence genes using conventional PCR technology among the APEC strains isolated from diseased broiler chicken.

Out of nine antibiotics tested, none of the antibiotic showed 100% effectiveness against the *E coli* strains. We found the highest 98% of *E. coli* isolates were resistant to ampicillin and the lowest 16% of *E. coli* isolates were resistant to amikacin. Cotrimoxazole, doxycycline hydrochloride, and ciprofloxacin account more than 60% resistivity among the tested *E. coli* isolates. These antibiotic resistivity patterns of *E. coli* strains are comparable with the previous studies [3, 7, 13, 23]. Conversely, aminoglycoside amikacin was the most effective against 84% of *E. coli* strains, which is close to the study published by Bist et al. [24]. Inappropriate utilization of different types of antibiotics in the poultry feed and for disease prevention is the common practice in Chitwan districts and other regions of Nepal. Previous data have showed the marked increase in veterinary antibiotic sales in Nepal [13, 25]. Indiscriminate use of the antibiotics exerts a selection pressure leading to the development of drugs resistance strain of bacteria. The antibiotic resistant patterns found in this study suggest a threatening situation of prevalence of the antibiotic resistant *E. coli* strains among broiler chickens in Chitwan district. Multiple antibiotic resistant patterns showed 94% of the isolates were resistant to the three or more antimicrobials. The high prevalent of multidrug resistance in *E. coli* have been reported in Bangladesh [3], China [26], and Korea [27]. The proportion of the isolates with MAR index greater than 0.2 is 94%, and less than or equal to 0.2 is 6%. MAR index value greater than 0.2 indicates high-risk sources of contamination, where several antibiotics may often use for the control of diseases [28]. This suggests the strong indication about an indiscriminative and abusive administration of multiple antibiotics for prophylaxis or infection. Such multi-drug resistances ultimately replace the drug sensitive microorganisms from the antibiotic saturated environment [29].

In this study, the frequency of eleven virulence genes and their role in the pathogenicity was evaluated among APEC and non-APEC strains. APEC strains are characterized by the possession of at least five virulence genes, which enable them to survive an extraintestinal life [1]. To the best of our knowledge, this is the first report in Nepal regarding the study of APEC virulence associated genes by PCR technique. We found the virulence associated genes were more frequently appeared among APEC strains compared to non-APEC strains. The detection rates of *iss*, *iucD, ompT, hlyF, iroN, astA,* and *iutA* among APEC isolates were relatively higher than the detection rates of corresponding genes in the non-APEC strains. High frequency of virulence genes *ompT, hlyF, iucD,* and *irp-2* were found in both the APEC and non-APEC isolates. In contrast, the virulence genes *iss, papC*, and *tsh* were not detected from the non-APEC isolates. The detection rate of *irp-2* and *cva/cvi* genes revealed that these genes were frequently distributed in both the APEC and non-APEC strains. The frequency of four genes *iss, ompT, hlyF,* and *iroN* were 100% among the APEC isolates (Table 2). The virulence genes *irp2* and *iucD*, both are related to iron acquisition system which demonstrated different detection frequency between the APEC and non-APEC isolates. Among the 45 APEC isolates, 97.8% showed presence of *iucD* genes, whereas, presence of *irp-2* gene was lower (73.3%). Due to the comparable detection rate, this study indicates the possession of *iucD* gene is important characteristics of both APEC and non-APEC isolates. The frequency of virulence genes detected in this study is comparable with other studies. Kwon et al. studied the presence of eight genes among 18 APEC strains and found the frequency of virulence genes as: *iss* (100%), *tsh* (94%), *vat* (89%), *iucD* (83%), *irp-2* (67%), *astA* (56%), *cva/cvi* (16%), and *papC* (11%) [30]. In another study, De Carli et al. has reported high frequency of virulence genes (*hlyF*, 100%; *iroN*, 98.8%; *ompT*, 100%; *iss,* 96.3%; and *iutA*, 81.5%) among the APEC strains [1]. The detection rate of *papC* is low (55.6%) among the APEC isolates and not detected among the non-APEC isolates. Due to the higher detection rates (80–90%), virulence genes *astA, iucD,* and *iutA* are considered as essential genes for pathogenicity. This study also revealed that there was no uniform and absolute combination of the virulence genes which can differentiate APEC and non-APEC strains of *E. coli.* Additionally, the detection of *iss*, *papC*, and *tsh* genes exclusively only among the APEC strains could be considered as important virulent factors for colibacillosis. The high prevalence of APEC strains of *E. coli* found in this study based on molecular investigation is the first report to reveal the severity of APEC strains in Chitwan district.

## Conclusions

This study showed high prevalence of multiple antimicrobials resistant *E. coli* and high frequency of virulence genes in APEC strains isolated from the colibacillosis suspected broiler chickens in Chitwan, Nepal. Regular screening and monitoring of the virulence genes associated with the antibiotic resistant APEC strains is essential for implementing intervention program to reduce risk of colibacillosis. A holistic approach is required for the prevention and the control of avian colibacillosis in Nepal and other regions of the country. This can be achieved with the active involvement and cooperation of farmers, hatchery operators, drug importers and marketers, veterinary and allied professionals, and government regulatory agencies.

## Additional file

Additional file 1: **Figure S1**. is provided as supplementary material in a separate additional file. (PDF 965 kb)

## Abbreviations

APEC: Avian pathogenic *Escherichia coli*
ATCC: American Type Culture Collection
CLSI: Clinical and Laboratory Standards Institute
EMB: Eosine methylene blue
ExPEC: Avian extraintestinal pathogenic *E.coli*
MAR: Multiple Antibiotic Resistance
NADIL: National Avian Disease Investigation Laboratory
NMEC: Neonatal meningitis *E. coli*
UPEC: Human uropathogenic *E. coli*
