# Correction to: Antibiotic resistance pattern and virulence genes content in avian pathogenic *Escherichia coli* (APEC) from broiler chickens in Chitwan, Nepal

**DOI:** 10.1186/s12917-018-1453-9

**Published:** 2018-05-22

**Authors:** Manita Subedi, Rebanta Kumar Bhattarai, Bhuminand Devkota, Sarita Phuyal, Himal Luitel

**Affiliations:** 1grid.460993.1Center for Biotechnology, Agriculture and Forestry University, Rampur, Chitwan Nepal; 2grid.460993.1Department of Veterinary Microbiology and Parasitology, Agriculture and Forestry University, Rampur, Chitwan Nepal

## Correction

The original article [1] contains errors in author panels and their contributions, errors in both the Methodology and the Results sections, and errors with respect to funding sources. The affected sections of the manuscript and their respective regions of corrected text can be viewed ahead:

## Authorship

The correct authors of the original article [1] can be viewed in the header of this manuscript.

## Methods

Sample collections, bacterial isolation and identification

Fifty liver samples were collected from 50 colibacillosis suspected broiler chickens which were attended from May 2016 to January 2017 for routine diagnosis at National Avian Disease Investigation Laboratory (NADIL) and Veterinary Teaching Hospital, Agriculture and Forestry University, Rampur, Nepal.

## Detection of virulence genes

Isolated *E. coli* strains were investigated for the presence of eleven virulence genes (*iutA*, *iss*, *papC*, *iucD*, *tsh*, *irp-2*, *ompT*, *hlyF*, *iron*, *cva/cvi*, and *astA*) which are associated with colibacillosis. For the detection of virulence genes, genomic DNA was extracted from pure cultures of *E. coli* grown overnight in the MacConkey agar at 37 °C by using the DNeasy Blood and Tissue Kit (Qiagen, catalogue no. 69506). The quality of genomic DNA was checked by gel electrophoresis and measuring absorbance at A260/A280 and A260/A230 ratios using the Quawell UV spectrophotometer (Q3000, USA). The conventional PCR was used to amplify the virulence genes. The primers used for amplification were those described previously (Table 1) [17, 18]. The PCR was performed in 25 μL volume containing 12.5 μL Hot start Taq 2X master mix (BioLab Inc., New England), 1 μL each primer (1 μM concentration), 2 μL (20 ng/μL optimized by dilution) DNA template, and 8.5 μL nuclease free water. The PCR amplifications were conducted in T100™ Thermal Cycler (Bio-Rad, USA) and the cycling conditions were identical for all the samples as follows: 94 °C for 4 min; 35 cycles of 30 s at 94 °C, 1 min at 60 °C, and 2 min at 68 °C; and 72 °C for 7 min. The amplicons were analyzed by agarose gel electrophoresis with 1.5% agarose gel (Sigma-Aldrich, A4718) prepared in 1× TBE buffer (Thermo Fisher Scientific, B52). All the PCR products were stained with ethidium bromide. After electrophoresis, the bands were visualized and photographed under UV light. The amplified product was considered to contain virulence gene if it produced band of the expected size.

**Table 1 Tab1:** Primer sets for detection of target virulence genes from avian pathogenic *Escherichia coli* (APEC) isolates

Genes	Primer Sequence (5′–3′)	Amplicon size (bp)
*iutA*	F: GGCTGGACATCATGGGAACTGG R: CGTCGGGAACGGGTAGAATCG	302
*iss*	F: CAGCAACCCGAACCACTTGATG R: AGCATTGCCAGAGCGGCAGAA	309
*papC*	F: TGATATCACGCAGTCAGTAGC R: CCGGCCATATTCACATAA	501
*iucD*	F: ACAAAAAGTTCTATCGCTTCC R: CCTGATCCAGATGATGCTC	714
*tsh*	F: ACTATTCTCTGCAGGAAGTC R: CTTCCGATGTTCTGAACGT	824
*irp-2*	F: AAGGATTCGCTGTTACCGGAC R: AACTCCTGATACAGGTGGC	413
*ompT*	F: TCATCCCGGAAGCCTCCCTCACTACTAT R: TAGCGTTTGCTGCACTGGCTTCTGATAC	496
*hlyF*	F: GGCCACAGTCGTTTAGGGTGCTTACC R: GGCGGTTTAGGCATTCCGATACTCAG	450
*iroN*	F: AATCCGGCAAAGAGACGAACCGCCT R:GTTCGGGCAACCCCTGCTTTGACTTT	533
*cva/cvi*	F: TGGTAGAATGTGCCAGAGCAAG R: GAGCTGTTTGTAGCGAAGCC	1181
*astA*	F: TGCCATCAACACAGTATATCC R: TCAGGTCGCGAGTGACGGC	116

## Results

A total of 50 *E. coli* strains were isolated from 50 liver swab samples of colibacillosis suspected broiler chickens. The antibiogram profile of *E. coli* isolates showed highest resistance to ampicillin (98%) and least resistance to amikacin (16%) (Fig. 1). Out of 50 *E. coli* isolates, 47 (94%) isolates were resistant to three or more antibiotics. The MAR index analysis showed 94% of *E. coli* isolates had MAR index value of > 0.2 and 6% had MAR index value of ≤0.2. The proportions of isolates with the MAR index values of 0.3, 0.4, 0.5, and 0.6 were 12% 20%, 22%, and 20%, respectively. There was no significant association of prevalence of antibiotic resistant strains with the type of *E. coli* strains (*P* > 0.05).

Based on the genetic criteria for the pathogenicity, isolates containing at least five virulence genes were considered as the APEC strains and isolates containing less than five virulence genes were considered as the avian non-pathogenic *Escherichia coli* (non-APEC) strains. Out of 50 *E. coli* isolates, 45 (90%) isolates were found to be APEC strains and 5 (10%) isolates were found to be non-APEC strains (Table 2). Among 50 *E. coli* isolates, 7 isolates contained all the eleven virulence genes, 14 isolates contained ten virulence genes, 15 isolates contained nine virulence genes, 5 isolates contained eight virulence genes, 2 isolates contained seven virulence genes, 2 isolates contained five virulence genes, 4 isolates contained 4 virulence genes, and 1 isolates contained 3 virulence genes.

## Declarations

### Funding

This work was supported financially by Directorate of Research and Extension (DOREX), Agriculture and Forestry University, Rampur, Chitwan, Nepal and National Agricultural Research Development Fund (NARDEF), Nepal.

### Authors’ Contributions

HL, BD and RKB conceived the concept, design, and supervised this study. MS and SP performed experimental work. MS, RKB and HL analyzed data and prepared the final draft of the manuscript. All authors read and approved the final manuscript.

## References

Reference [24] in the original article mistakenly omits some author details and as such, the correct presentation of this reference can be seen in reference [2] of this Correction article.
