# Supplementary material for: Antibiotic resistance pattern and virulence genes content in avian pathogenic Escherichia coli (APEC) from broiler chickens in Chitwan, Nepal
Source: BMC Vet Res. 2018 Mar 27;14:113. doi: 10.1186/s12917-018-1442-z (PMC5870509; doi:10.1186/s12917-018-1442-z)
Supplement: Supplementary file 1 — Figure S1. is provided as supplementary material in a separate additional file. (PDF 965 kb) [file 12917_2018_1442_MOESM1_ESM.pdf]

Additional file 1

BMC Veterinary Research

Antibiotic resistance pattern and virulence genes content in avian pathogenic *Escherichia coli* (APEC) from broiler chickens in Chitwan, Nepal

Manita Subedi[1], Himal Luitel[2], Bhuminanda Devkota[2], Rebanta Kumar Bhattarai[3], Sarita Phuyal[4], Prabhat Panthi[5], Anil Shrestha[6] and Dhiraj Kumar Chaudhary[7*]

[1]Department of Drug Administration, Government of Nepal, Bijulibazar, Kathmandu, Nepal

[2]Center for Biotechnology, Agriculture and Forestry University, Rampur, Chitwan, Nepal

[3]Department of Veterinary Microbiology and Parasitology, Agriculture and Forestry University, Rampur, Chitwan, Nepal

[4]Department of Veterinary Science & Animal Husbandry, Himalayan College of Agriculture Sciences and Technology, Kathmandu, Nepal

[5]Department of Microbiology, National College, Kathmandu, Nepal

[6]Department of Microbiology, Balkumari College, Chitwan, Nepal

[7]Department of Microbiology, Prithu Technical College, Institute of Agriculture and Animal Science, Tribhuvan University, Dang, Nepal

Content category: Research article

***Corresponding author:** Dhiraj Kumar Chaudhary

Tel: +977-9841441236

E-mail: dhirajchaudhary2042@gmail.com

Additional Figure S1: Virulence genes detection from *E. coli* strains isolated from colibacillosis suspected broiler chickens. a, *iutA*; b, *iss*; c, *papC*; d, *iucD*; e, *tsh*; f, *irp-2*; g, *ompT*; h, *hlyF*; i, *iron*; j, *cva/cvi*; k, *astA*. M: 1Kb DNA ladder; Lane 1–50: *E. coli* isolates.

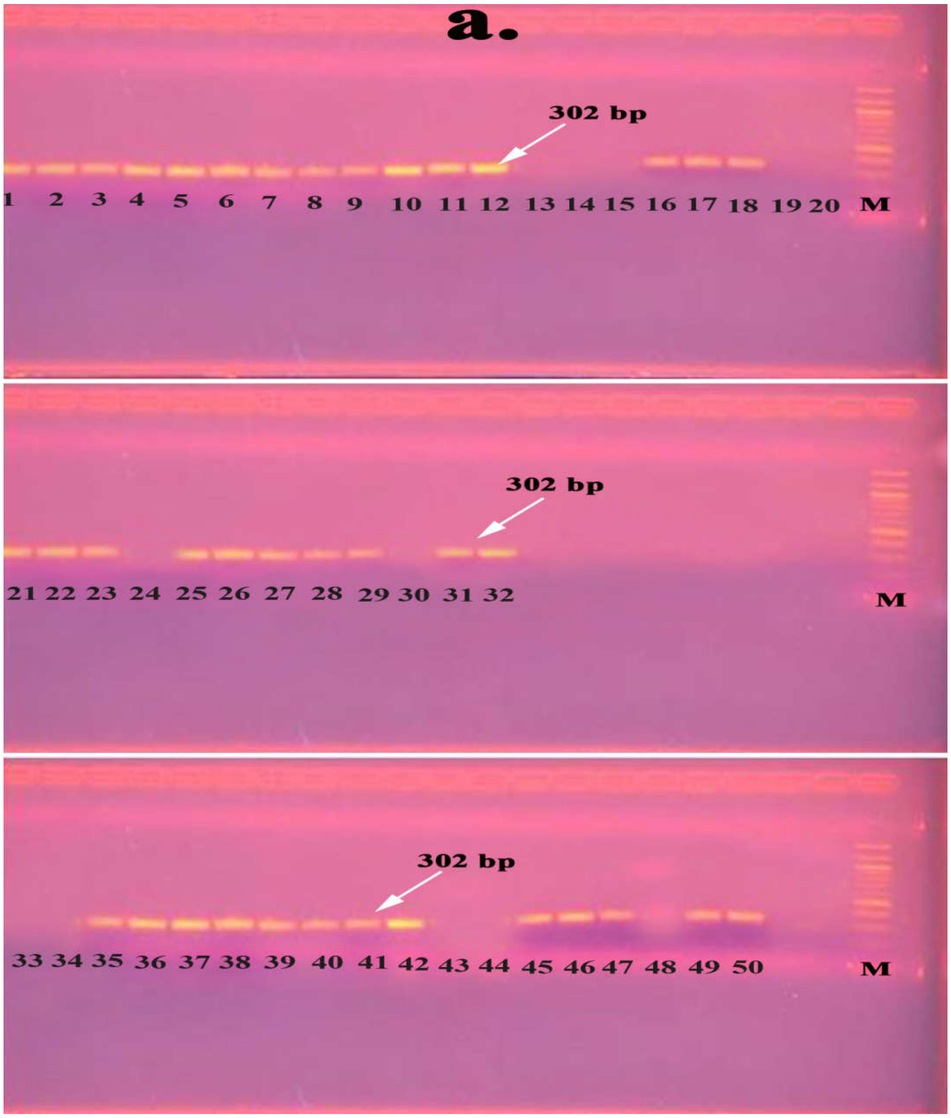

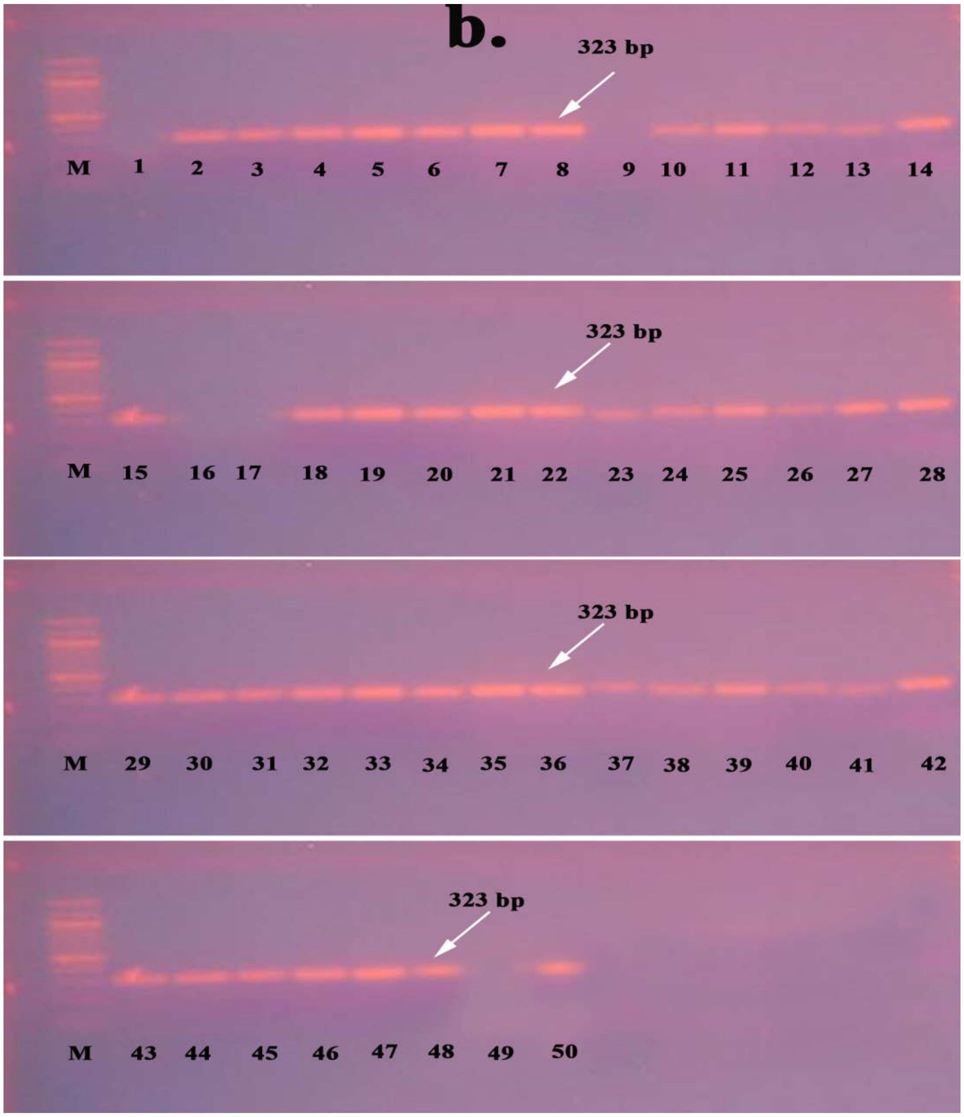
b.
323 bp
M 1 2 3 4 5 6 7 8 9 10 11 12 13 14
323 bp
M 15 16 17 18 19 20 21 22 23 24 25 26 27 28
323 bp
M 29 30 31 32 33 34 35 36 37 38 39 40 41 42
323 bp
M 43 44 45 46 47 48 49 50

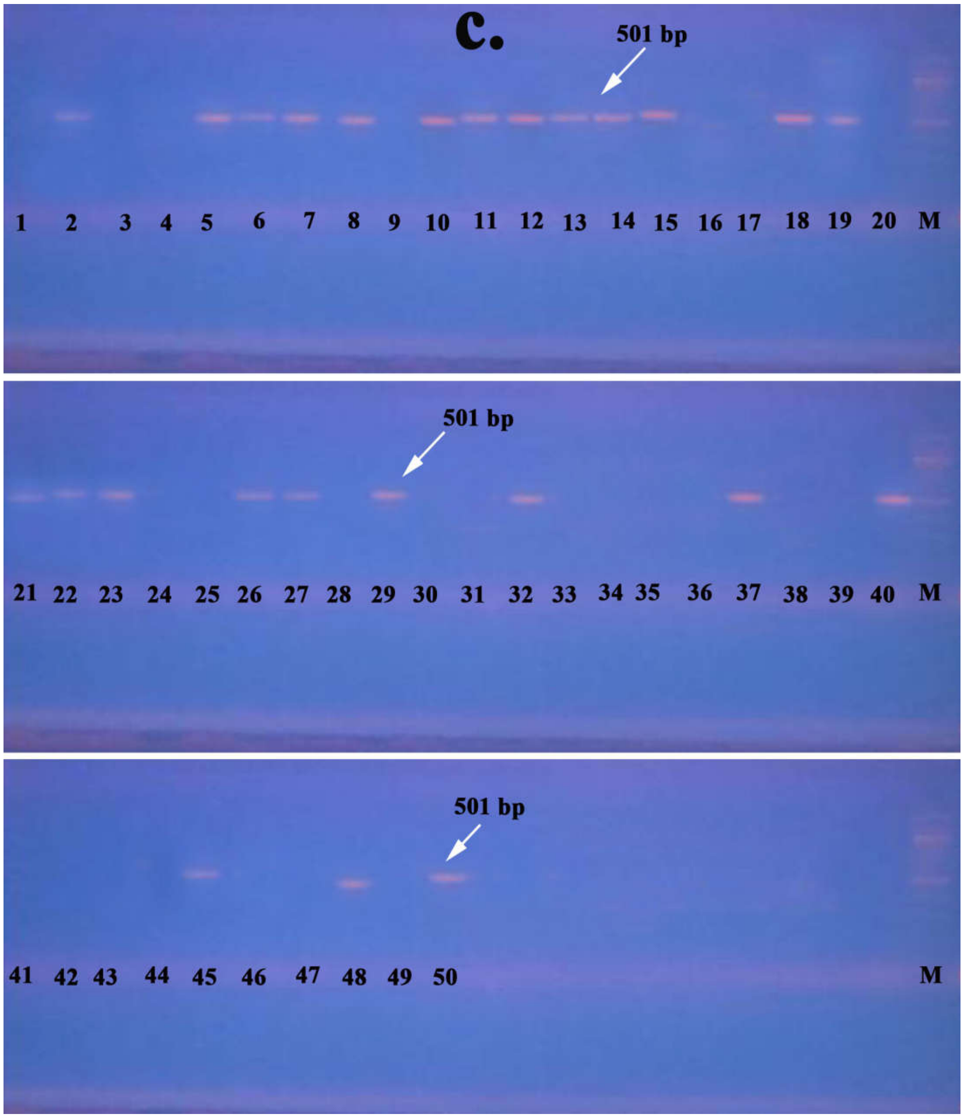
c.
501 bp
1 2 3 4 5 6 7 8 9 10 11 12 13 14 15 16 17 18 19 20 M
501 bp
21 22 23 24 25 26 27 28 29 30 31 32 33 34 35 36 37 38 39 40 M
501 bp
41 42 43 44 45 46 47 48 49 50 M

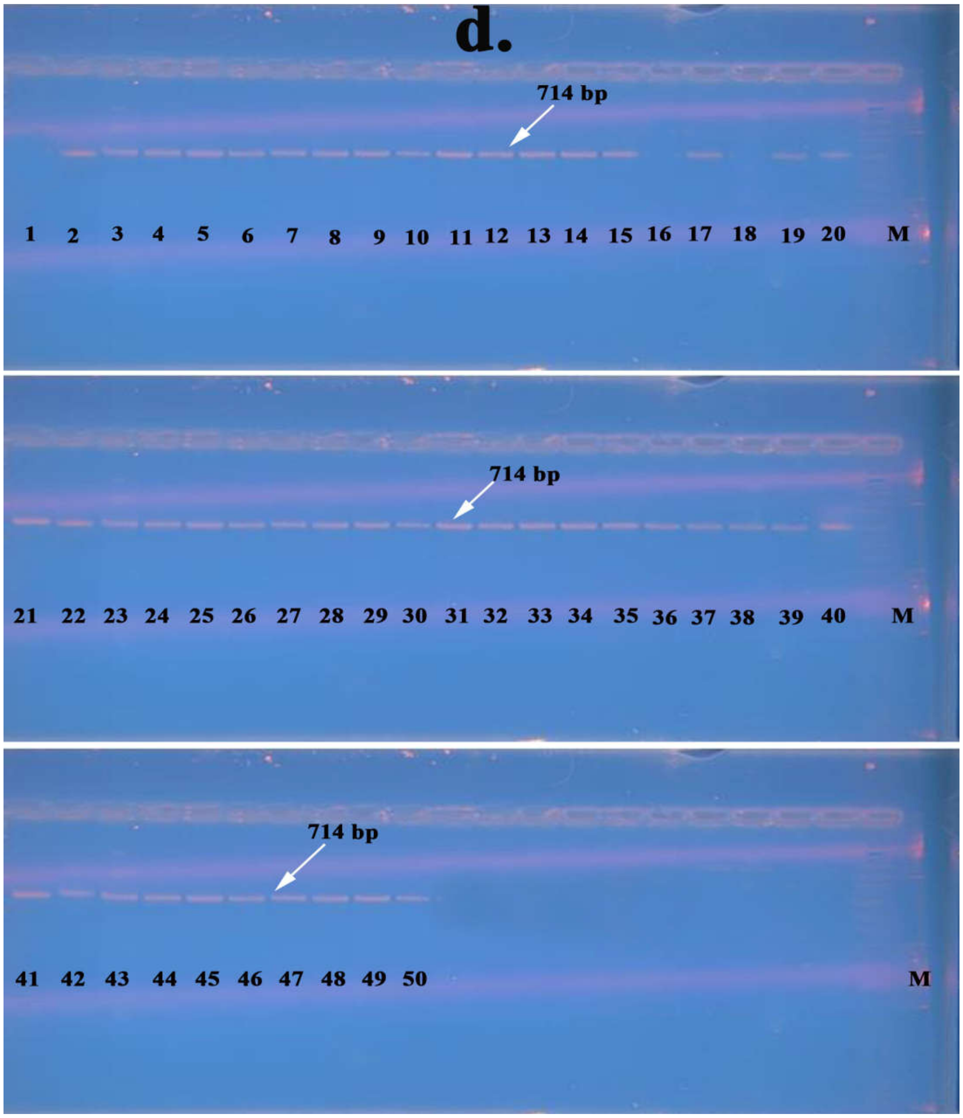
d.
714 bp
1 2 3 4 5 6 7 8 9 10 11 12 13 14 15 16 17 18 19 20 M
714 bp
21 22 23 24 25 26 27 28 29 30 31 32 33 34 35 36 37 38 39 40 M
714 bp
41 42 43 44 45 46 47 48 49 50 M

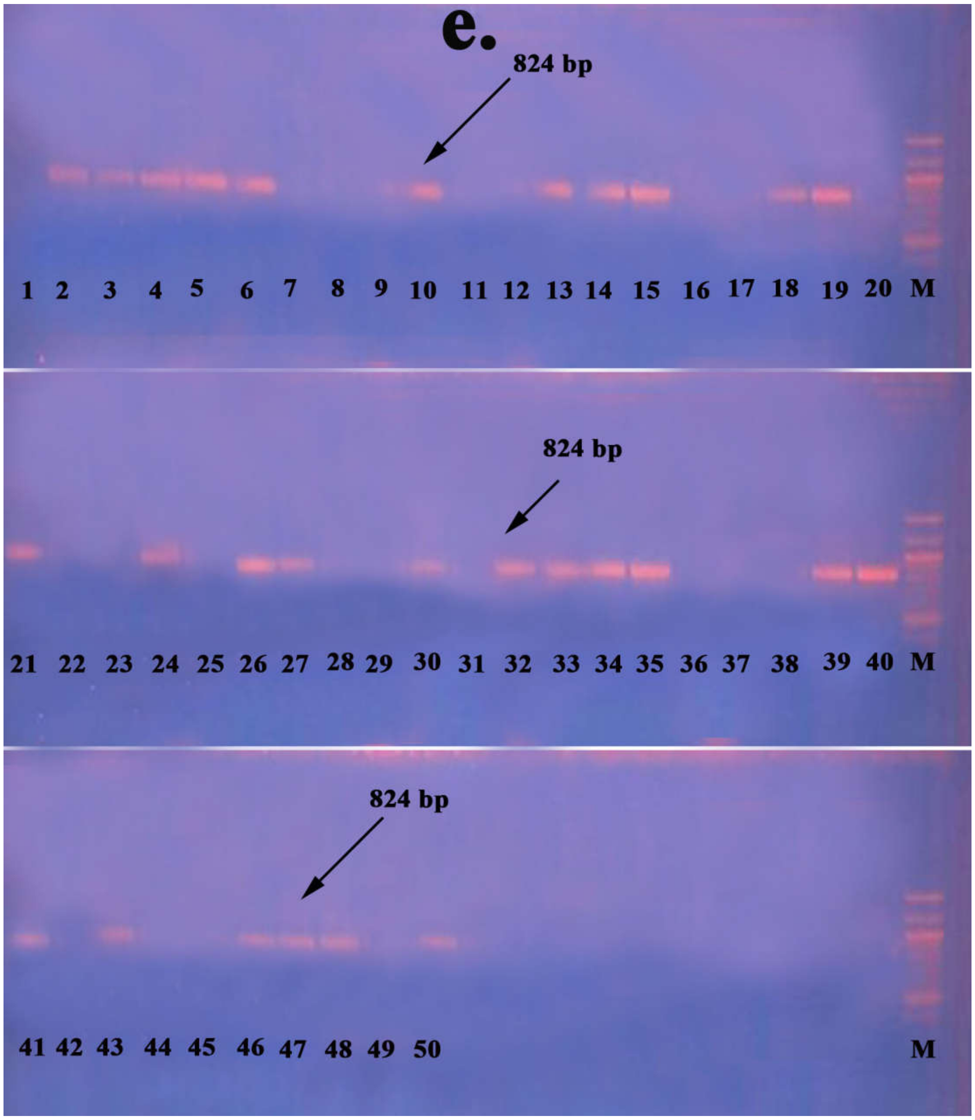
e.
824 bp
1 2 3 4 5 6 7 8 9 10 11 12 13 14 15 16 17 18 19 20 M
824 bp
21 22 23 24 25 26 27 28 29 30 31 32 33 34 35 36 37 38 39 40 M
824 bp
41 42 43 44 45 46 47 48 49 50 M

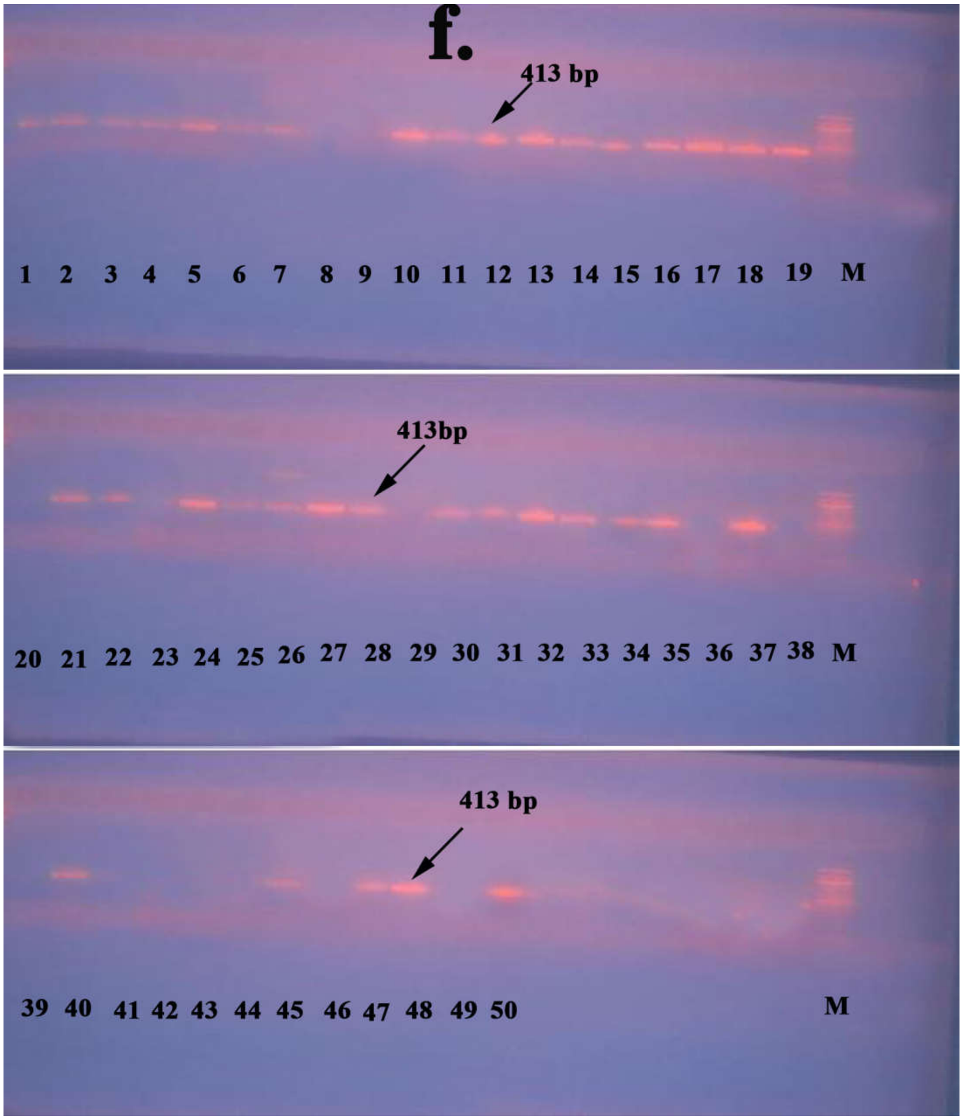
f.
413 bp
1 2 3 4 5 6 7 8 9 10 11 12 13 14 15 16 17 18 19 M
413bp
20 21 22 23 24 25 26 27 28 29 30 31 32 33 34 35 36 37 38 M
413 bp
39 40 41 42 43 44 45 46 47 48 49 50 M

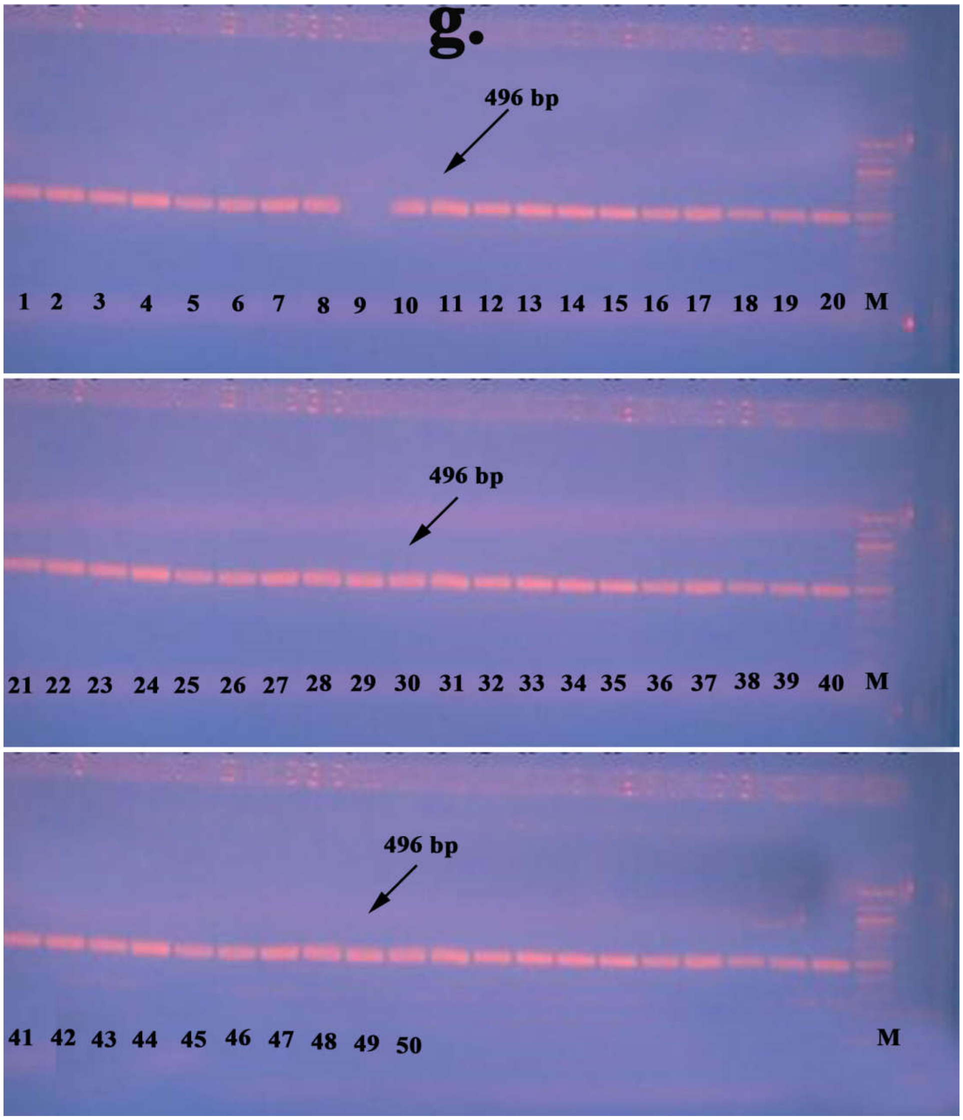
g.
496 bp
1 2 3 4 5 6 7 8 9 10 11 12 13 14 15 16 17 18 19 20 M
496 bp
21 22 23 24 25 26 27 28 29 30 31 32 33 34 35 36 37 38 39 40 M
496 bp
41 42 43 44 45 46 47 48 49 50 M

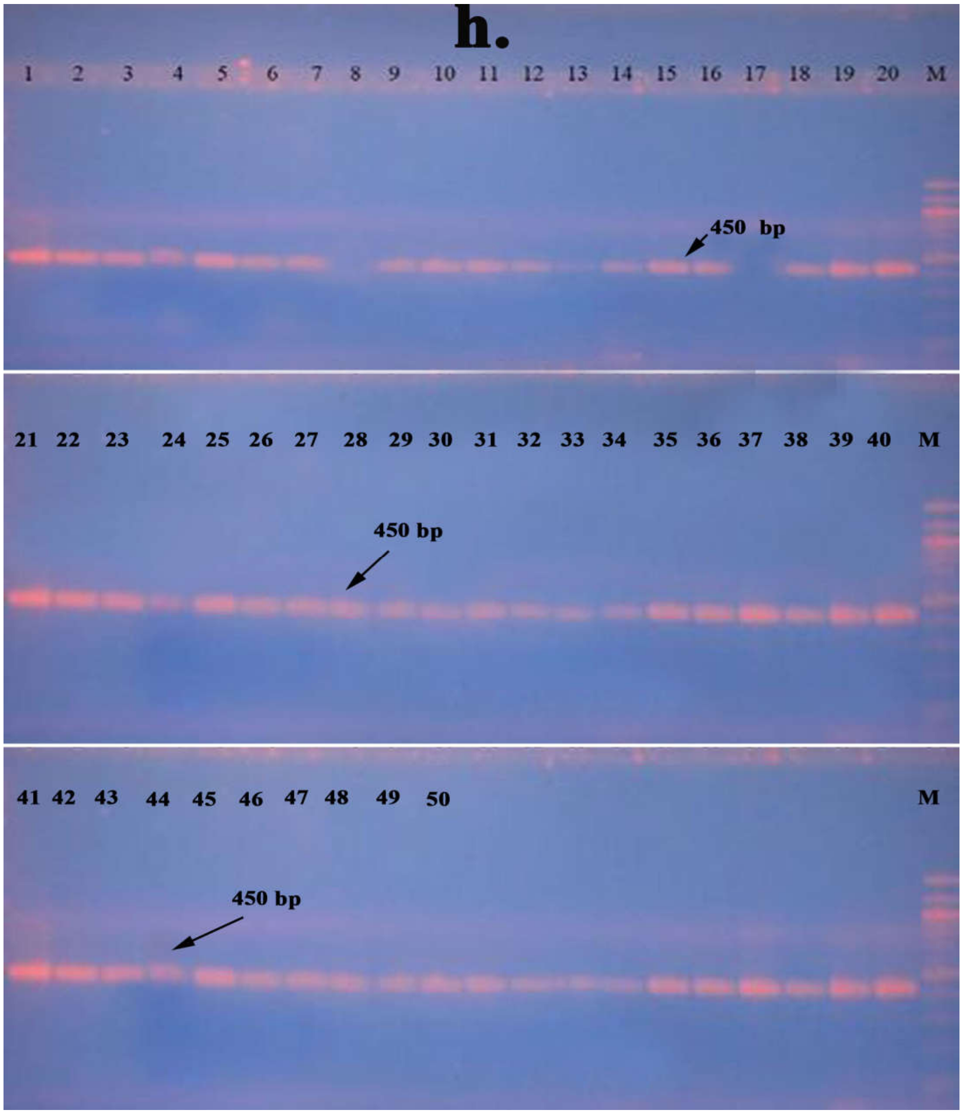
h.
1 2 3 4 5 6 7 8 9 10 11 12 13 14 15 16 17 18 19 20 M
450 bp
21 22 23 24 25 26 27 28 29 30 31 32 33 34 35 36 37 38 39 40 M
450 bp
41 42 43 44 45 46 47 48 49 50 M
450 bp

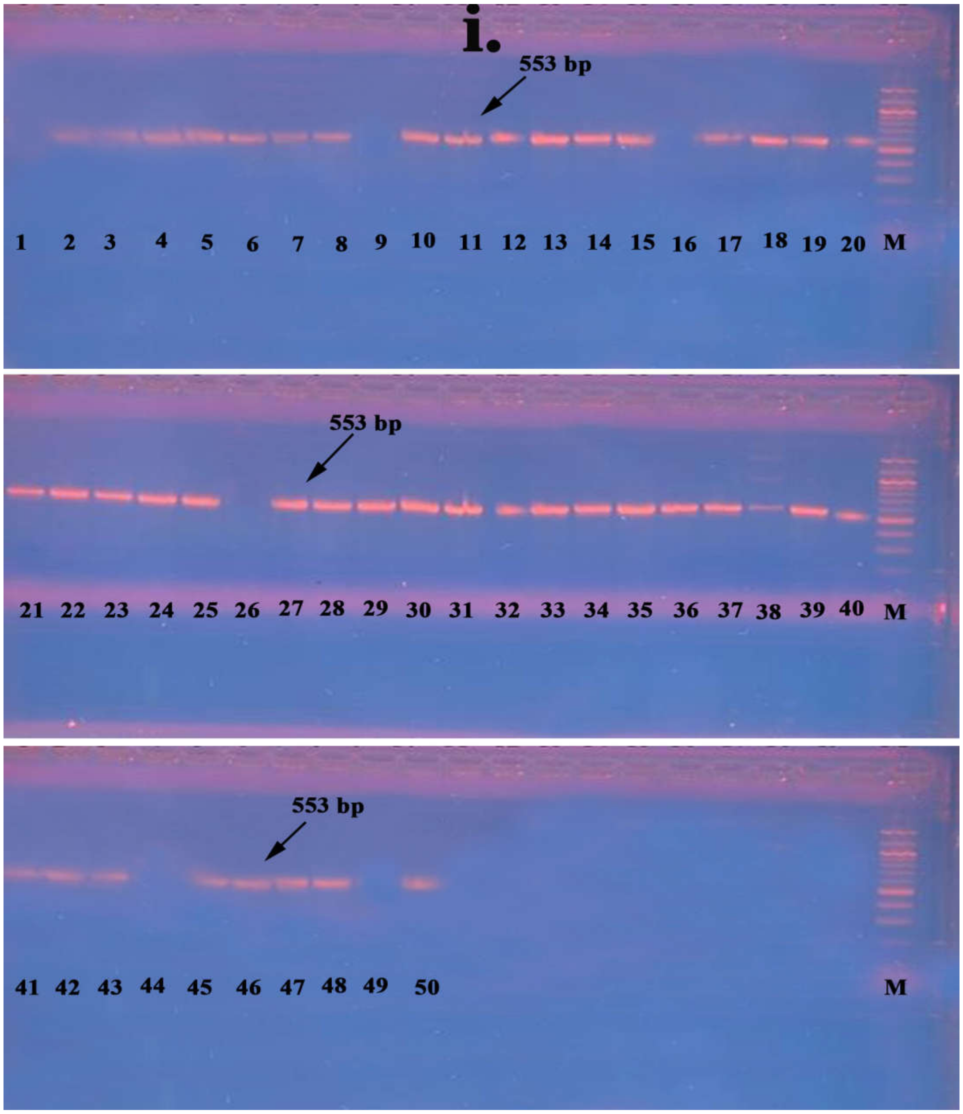
i.
553 bp
1 2 3 4 5 6 7 8 9 10 11 12 13 14 15 16 17 18 19 20 M
553 bp
21 22 23 24 25 26 27 28 29 30 31 32 33 34 35 36 37 38 39 40 M
553 bp
41 42 43 44 45 46 47 48 49 50 M

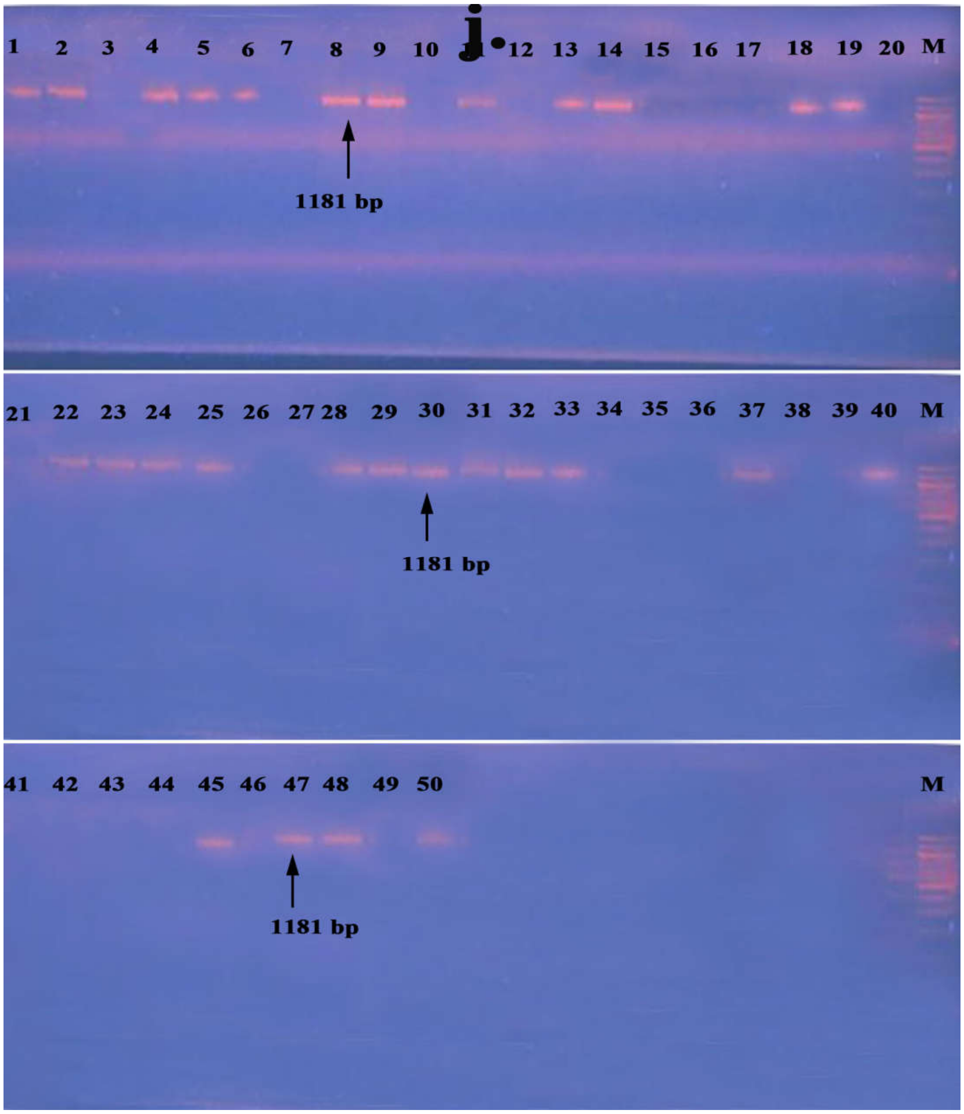
j.
1 2 3 4 5 6 7 8 9 10 11 12 13 14 15 16 17 18 19 20 M
1181 bp
21 22 23 24 25 26 27 28 29 30 31 32 33 34 35 36 37 38 39 40 M
1181 bp
41 42 43 44 45 46 47 48 49 50 M
1181 bp

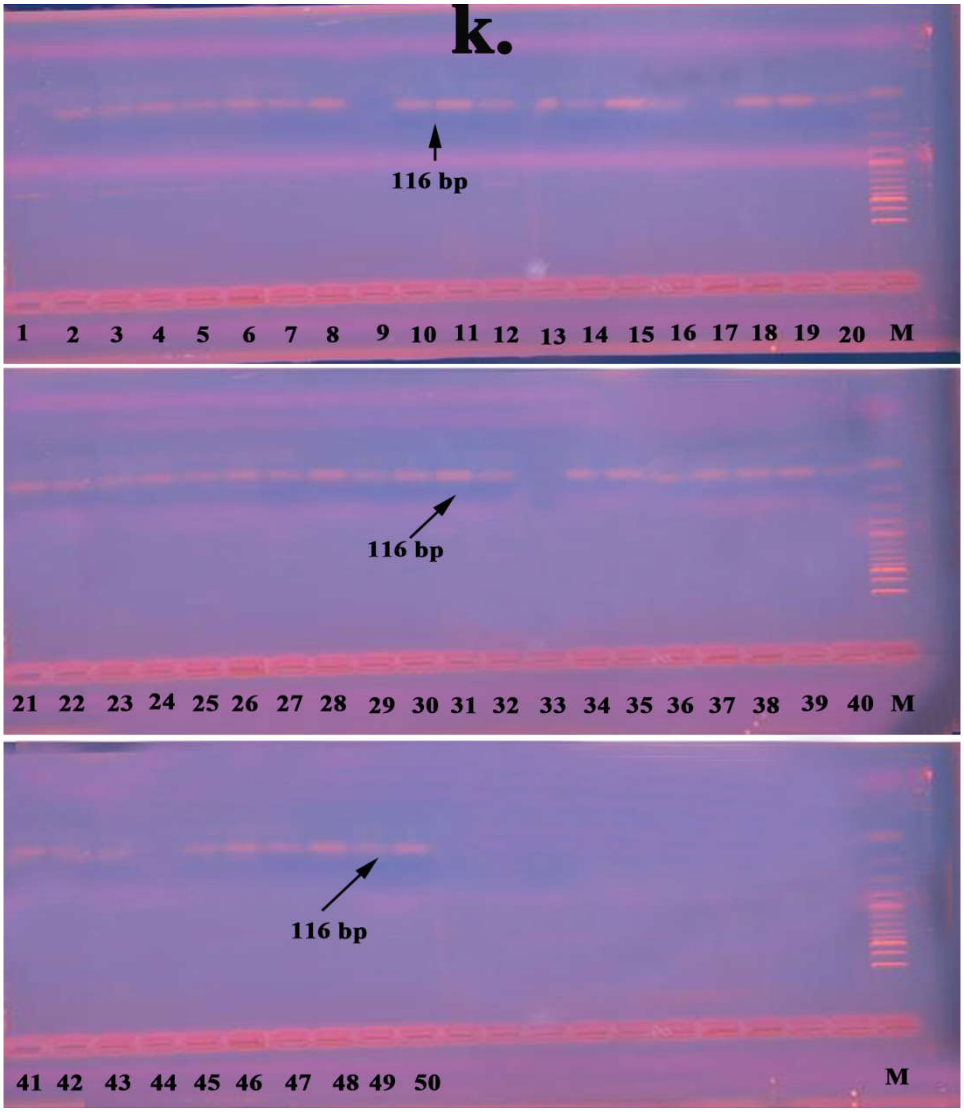
k.
116 bp
1 2 3 4 5 6 7 8 9 10 11 12 13 14 15 16 17 18 19 20 M
116 bp
21 22 23 24 25 26 27 28 29 30 31 32 33 34 35 36 37 38 39 40 M
116 bp
41 42 43 44 45 46 47 48 49 50 M
